# Significant perioperative parameters affecting postoperative complications within 30 days following craniotomy for primary malignant brain tumors

**DOI:** 10.1186/s13741-023-00343-x

**Published:** 2023-10-23

**Authors:** Yao-Chung Yang, Yao-Shen Chen, Wei-Chuan Liao, Chun-Hao Yin, Yung-Shang Lin, Meng-Wei Chen, Jin-Shuen Chen

**Affiliations:** 1 Division of Neurosurgery, Department of Surgery, Kaohsiung Veterans General Hospital, Kaohsiung, Taiwan; 2 Department of Biological Sciences, National Sun Yat-Sen University, Kaohsiung, Taiwan; 3 Department of Administration, Kaohsiung Veterans General Hospital, Kaohsiung City, 81362 Taiwan; 4 Department of Medical Education and Research, Kaohsiung Veterans General Hospital, Institute of Health Care Management, National Sun Yat-Sen University, Kaohsiung, Taiwan; 5 Department of Surgery, Kaohsiung Armed Force General Hospital, Kaohsiung, Taiwan

**Keywords:** Glomerular filtration rate, Primary malignant brain tumor, Postoperative complication

## Abstract

**Background:**

The occurrence of postoperative complications within 30 days (PC1M) of a craniotomy for the removal of a primary malignant brain tumor has been associated with a poor prognosis. However, it is still unclear to early predict the occurrence of PC1M. This study aimed to identify the potential perioperative predictors of PC1M from its preoperative, intraoperative, and 24-h postoperative parameters.

**Methods:**

Patients who had undergone craniotomy for primary malignant brain tumor (World Health Organization grades III and IV) from January 2011 to December 2020 were enrolled from a databank of Kaohsiung Veterans General Hospital, Taiwan. The patients were classified into PC1M and nonPC1M groups. PC1M was defined according to the classification by Landriel et al. as any deviation from an uneventful 30-day postoperative course. In both groups, data regarding the baseline characteristics and perioperative parameters of the patients, including a new marker-kinetic estimated glomerular filtration rate, were collected. Logistic regression was used to analyze the predictability of the perioperative parameters.

**Results:**

The PC1M group included 41 of 95 patients. An American Society of Anesthesiologists score of > 2 (*aOR*, 3.17; 95% confidence interval [CI], 1.19–8.45; *p* = 0.021), longer anesthesia duration (*aOR*, 1.16; 95% *CI*, 0.69–0.88; *p* < 0.001), 24-h postoperative change in hematocrit by >  − 4.8% (*aOR*, 3.45; 95% *CI*, 1.22–9.73; *p* = 0.0019), and 24-h postoperative change in kinetic estimated glomerular filtration rate of < 0 mL/min (*aOR*, 3.99; 95% *CI*, 1.52–10.53; *p* = 0.005) were identified as independent risk factors for PC1M via stepwise logistic regression analysis. When stratified according to the age of ≥ 65 years (*OR*, 11.55; 95% *CI*, 1.30–102.79; *p* = 0.028), the reduction of kinetic estimated glomerular filtration rate was more robustly associated with a higher risk of PC1M.

**Conclusions:**

Four parameters were demonstrated to significantly influence the risk of PC1M in patients undergoing primary malignant brain tumor removal. Measuring and verifying these markers, especially kinetic estimated glomerular filtration rate, would help early recognition of PC1M risk in clinical care.

## Background

The annual incidence of primary malignant brain tumors (PMBTs) is approximately 4–7 per 100,000 people in the USA (Ostrom et al., 2020). Although therapeutic protocols have considerably improved over time, PBMTs continue to engender substantial morbidity and mortality. Generally, the primary therapeutic approaches of PMBTs include surgery, radiotherapy, chemotherapy, or target therapy alone or in various combinations. The typical surgical treatment involves tumor removal via craniotomy, which is becoming increasingly refined to maximize the amount of tumor removed; however, offering survival benefits while preserving neurological function remains challenging.

Broadly, short-term postoperative complications can be categorized into neurological, regional, and systemic events, including direct cortical and vascular injuries, surgical wound complications, and postsurgical medical complications. Most studies consider 30 days as the short-term postoperative period for evaluating outcomes and complications (Lassen et al., 2011; Senders et al. 2018). Such complications substantially contribute to increased mortality and neurological decline, frequently leading to extended hospitalization, reoperation, readmission, and treatment delay of adjuvant chemotherapy and radiotherapy (Cinotti et al. 2018; Lonjaret et al. 2017; Senders et al. 2018; Zhang et al. 2021). Thus, predicting short-term complications following craniotomy for PMBTs and ensuring early treatment for the same remain challenging.

Understanding and reducing postoperative complications within 30 days (PC1M) will help generate interventions focused on reducing the burden of these adverse outcomes. Numerous factors may predict the development of PC1M following a craniotomy for primary malignant brain tumors (PMBT). These factors can be grouped into patient-related, surgery-related, and anesthesia-related categories (Harland et al. 2020; Hurtado et al., 2020; Lonjaret et al. 2017; Missios et al. 2015). However, to the best of our knowledge, the perioperative changes of serum laboratory parameters and renal clearance have not been considered potential predictors of PC1M development. The perioperative period includes the three phases of surgery: preoperative, intraoperative, and postoperative phases. The kidneys and the brain are crucial for maintaining homeostasis and extracellular fluid balance by controlling the renal blood flow and glomerular filtration rate (GFR), thereby influencing renal sodium handling (Davenport 2008). Previous research has focused predominantly on renal dysfunction induced by traumatic brain injury (Freeman & Wadei 2015); the impact of kidney function in a postcraniotomy status remains relatively unexplored, particularly as potential predictors of postoperative outcomes. Furthermore, postoperative renal clearance may rapidly change due to the distribution of body fluid volume, thus making the assessment of the glomerular filtration rate (eGFR) challenging. The kinetic eGFR (KeGFR) formula, developed by S. Chen, considers the time interval between rising creatinine levels and the volume of distribution to evaluate GFR during the acute phase accurately. We expected our study to clarify these predictors.

This study had two primary objectives: to evaluate the effect of perioperative parameters—parameters within 7 days before surgery, intraoperative surgical metrics, parameters within 24 h after surgery, and perioperative changes in serum laboratory parameters—on PC1M following craniotomy for tumor resection and to estimate the perioperative changes in renal clearance according to the 4 variable Modification of Diet in Renal Disease (MDRD) equation and KeGFR equation and compare the values of the two methods for predicting PC1M.

## Methods

### Study population and variable collections

**Fig. 1:**
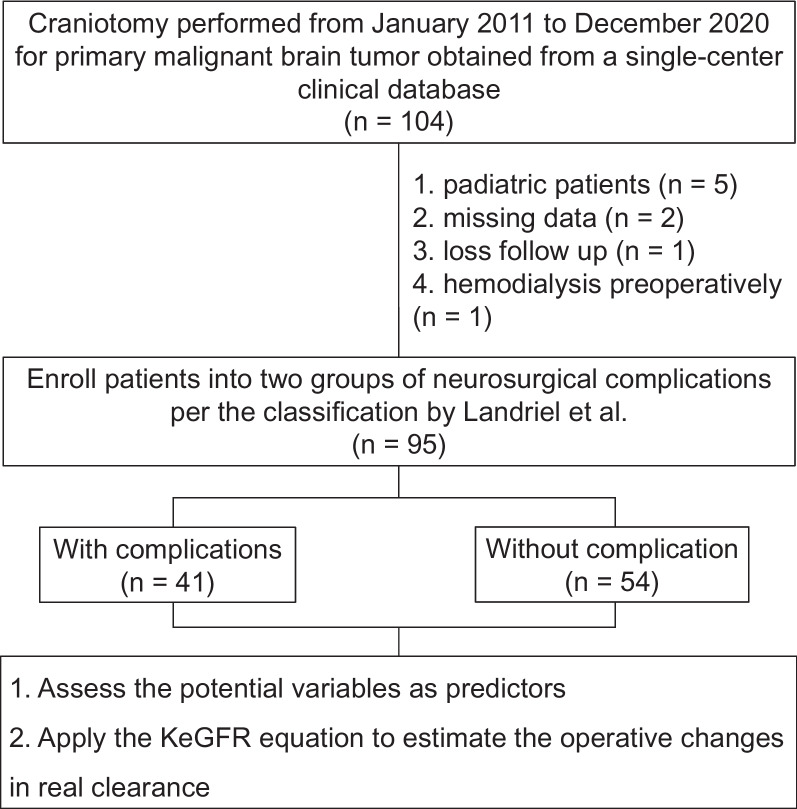
Flow diagram summarizing the patients included in the study and the parameters obtained. Abbreviation: *KeGFR* Kinetic estimated glomerular filtration rate

Herein, we conducted a retrospective observational study of adult patients who underwent craniotomy for PMBT (World Health Organization (WHO) grades III or IV) between January 2011 and December 2020 in Kaohsiung Veterans General Hospital (KVGH), Kaohsiung, Taiwan. We routinely use cefazolin as perioperative antibiotics, with one dose administered preoperatively, additional doses given every 4 h during surgery, and then every 8 h postoperatively, for a total duration of 24 h. This procedure is in accordance with the surgical antibiotic prophylaxis guidelines. There was no association between prophylactic and therapeutic antibiotic use. Patients aged < 20 years, with chronic renal failure who had undergone long-term dialysis, previously undergone brain surgery, chemotherapy, or radiotherapy, and patients with incomplete clinical data during the perioperative period were excluded. Each PC1M was recorded according to the classification by Landriel et al. as any deviation from an uneventful postoperative course within 30 days after surgery (Landriel Ibañez et al., 2011). The Landriel-Ibañez classification system assessed postoperative complications within 30 days of neurosurgical procedures. This classification grades the complications on a four-grade scale: Grade I represents any non-life-threatening complication managed without invasive procedures, Grade II refers to complications requiring invasive management, Grade III signifies life-threatening events that necessitate treatment in an intensive care unit, and Grade IV denotes death resulting from complications. We further categorized each grade as either surgical or medical complications. This systematic classification allowed us to compare the severity of complications among patients, the incidence of surgical versus medical complications, and the progression of complications over time. The patients were classified according to the occurrence of PC1M (PC1M and nonPC1M groups). The flowchart of patient selection is shown in Fig. 1.

To obtain the baseline demographics, clinical characteristics, and perioperative parameters, the electronic medical records and charts of the patients were reviewed manually. The perioperative data included the 7-day preoperative serum parameters, intraoperative surgical metrics, and 24-h postoperative serum parameters. The health status of the patients were evaluated before surgery according to several scoring systems. Frailty was determined using the modified 5-item frailty index (mFI-5), which was manually calculated by tracking the presence or absence of the following five medical comorbidities: functional status, history of diabetes mellitus, chronic obstructive pulmonary disease, congestive heart failure, and hypertension. The presence of each comorbidity corresponds to 1 point. Thus, the minimum and maximum scores are 0 and 5, respectively. The volume of the tumor was measured via preoperative postcontrast magnetic resonance images, following the abc/2 technique for estimation. The American Society of Anesthesiologists (ASA) Physical Status Classification System score, ranging in increments of 1 from a localized disease not engendering systemic disturbance (1) to extreme systemic disorders and a poor physical state (5), was documented for each patient. Preoperative laboratory testing is optimal immediately before surgery; however, it is often performed earlier for practicality. According to previous studies, blood test results as early as 2 months before surgery and those obtained immediately before surgery demonstrate no differences in the risk of 30-day morbidity and mortality (Ruetzler et al. 2018). We considered testing within 7 days before surgery reasonable to provide a baseline biochemistry profile of the patients. The following preoperative serum laboratory parameters were assessed within 7 days before surgery: white blood cell (WBC) count, hematocrit (Hct), hemoglobin level, platelet count, creatinine level, blood urea nitrogen level, estimated GFR, and sodium, potassium, and glutamate pyruvate transaminase levels. The vital signs of the patients were recorded during surgery, including their intraoperative body temperature, blood pressure, heart rate, blood loss, and anesthesia duration. Anesthesia duration was defined as the time from anesthesia induction preparation by the anesthesiologist to the cessation of narcotic drug use. Routine postoperative serum laboratory tests were performed within 24 h following surgery to compare the results with the preoperative values. The perioperative mean change was defined as the difference between the preoperative and postoperative laboratory data. Furthermore, we performed subgroup analyses to determine the robustness of the association between renal clearance change and PC1M risk. We selected and stratified the patients by age and sex, which are the major influencing factors of physiological renal function loss. As the serum creatinine level gradually increases in response to a single-step change in GFR, a new steady-state creatinine-based estimate of GFR requires 24–72 h to equilibrate the creatinine plasma levels. Until this new equilibrium develops, staging criteria relying on the peak creatinine value cannot be used to predict postoperative outcomes. Therefore, the KeGFR equation was used herein, which calculated the 24-h postoperative creatinine value to correctly reflect the actual GFR (Endre et al. 2011).

### Equations for estimating renal clearance change

Two calculation models were used herein. The preoperative serum creatinine level in the 4 variable MDRD equation was used to derive a preoperative estimated GFR (Levey et al. 2006).

$$\mathrm{GFR }= 175 \times \left(\mathrm{sCr}\right)-1.154 \times \left(\mathrm{age}\right)-0.203 \times (0.742\mathrm{ if female}) \times (1.212\mathrm{ if African American})$$

Postoperative renal clearance was calculated using both the 4 variable MDRD and KeGFR equations. To calculate KeGFR, an estimate of creatinine production over time was used, validated with patients with varying degrees of kidney function. The KeGFR equation, created by Chen, requires two different creatinine values obtained at different times (we used the preoperative and postoperative creatinine values) (Chen 2013).

$$KeGFR = ([bCr \times CrCl]/mean sCr) \times (1-[\{24 \times \Delta sCr\}/\{\Delta t(h) \times Max\Delta sCr/day\}])$$

where bCr denotes the baseline serum creatinine level (because kidney function in the patients in this study was relatively healthy, we defined bCr as the preoperative creatinine level), CrCl denotes the corresponding creatinine clearance (preoperative value), mean sCr denotes the average of the two serum creatinine values, ΔsCr denotes the difference between the two serum creatinine values, Δt denotes the difference between the two creatinine collecting times in hours, and MaxΔsCr/day denotes the maximal increase in serum creatinine level per day assuming no renal function. In previous studies, the serum creatinine level usually ranged from 1.3 to 2.0 mg/dL per day in patients on dialysis. In accordance with the study by Chan et al., we used a consistent default value of 1.5 for MaxΔsCr/day for most adult patients, which was not extensively inaccurate.

KeGFR was derived from the initial creatinine content, volume of distribution, creatinine production rate, and quantitative changes in consecutive plasma creatinine levels before and after surgery. Therefore, the change in GFR was calculated as two independent numbers via two methods: (1) postoperative KeGFR - preoperative estimated GFR from the MDRD equation and (2) postoperative estimated GFR − preoperative estimated GFR from the MDRD equation.

### Statistical analysis

The normality of baseline demographics and clinical characteristics was assessed with the Kolmogorov–Smirnov test. Parametric continuous variables are presented as mean and standard deviation and were analyzed via one-way ANOVA. Nonparametric data, expressed as median and interquartile range, were subjected to independent Mann–Whitney *U*-tests. Categorical variables between the groups, expressed as number (percentage), were compared using the chi-square test. In cases where the expected values were less than 3, Fisher’s exact test was employed. The mean changes in serum laboratory parameters during the perioperative period were incorporated as binary factors into the logistic regression analysis. Backward selection process began with the univariable analysis of the collected parameters. In the multivariable analysis, any variable in the univariable test with a *p*-value of < 0.1 was used (Bursac et al. 2008). An algorithm involving logistic regression was employed to assess variables associated with neurosurgical complications, accounting for baseline characteristics, preoperative laboratory parameters, intraoperative surgical metrics, postoperative laboratory parameters, and perioperative changes in laboratory parameters, to identify potential predictive factors. Additionally, to identify risk factors for PC1M, we performed stratification analysis to measure renal clearance change and determine which population considering sex and age was most susceptible to PC1M.

We used SAS version 9.4 (SAS System for Windows; SAS Institute, Cary, NC, USA) and SPSS Statistics 20 (IBM Corporation, Armonk, NY, USA) to perform all statistical analyses. A *p*-value of < 0.05 was considered statistically significant.

### Ethical issues

The ethics committee of KVGH approved the study (IRB permit number KSVGH21-CT5-20). The requirement for patient consent was waived because of the retrospective and observational design of the study and no increase in the health risk of any patient.

## Results

### Comprehensive demographic data and perioperative characteristics of the PC1M and nonPC1M groups

The baseline characteristics of the 95 patients who underwent craniotomy for a PMBT are listed in Table 1. The mean age was 55 ± 16 years, and 55% of the patients were men. Glioblastomas were the most commonly occurring tumors (73.7%), followed by anaplastic astrocytomas (10.5%) and anaplastic oligodendrogliomas (8.4%); the mean tumor size was 6.7 ± 1.7 cm^3^. Classified using the WHO grading system, all malignancies correspond to WHO grades III or IV. Among these, 72 patients, representing 76%, are categorized as WHO grade IV. In the PC1M group, 18 (43.9%) and 23 (56.1%) were classified as surgical and medical complications, respectively, per the classification by Landriel et al. Furthermore, 56.1%, 9.6%, 29.3%, and 4.9% of the complications were grade I (mild), grade II (moderate), grade III (severe), and grade IV (death), respectively (Table 2). The mFI-5 scores, tumor characteristics, and preoperative seizure occurrence did not differ significantly between the PC1M and nonPC1M groups. Conversely, the KeGFR equation yielded significant different values for perioperative renal clearance changes for the nonPC1M (0.9 ± 6.3 mL/min) and PC1M (− 2.0 ± 7.1 mL/min; Table 3) groups (*p* = 0.041). In univariable analysis, two preoperative physical factors, one intraoperative surgical metric, one postoperative parameter, and two perioperative serum changes differed significantly between the PC1M and nonPC1M groups: (1) lower preoperative GCS score (*p* = 0.018), (2) ASA physical status score of > 2 (*p* = 0.012), (3) longer anesthesia duration (*p* = 0.062), (4) decrease in 24-h postoperative blood urea nitrogen by > 0.3 mg/dL from the preoperative value (*p* = 0.097), (5) decrease in 24-h postoperative hematocrit levels by ≥ 4.8% from the preoperative value (*p* = 0.04), and (6) decrease in 24-h postoperative KeGFR value from the preoperative value (*p* = 0.01). Notably, the MDRD equation for the 24-h postoperative renal clearance indicated increase in both the nonPC1M (16.8 ± 30.9 mL/min) and PC1M (8.8 ± 23.1 mL/min) groups (*p* = 0.167).

**Table 1:** Baseline demographics and clinical characteristics of the patients who underwent craniotomy for primary malignant brain tumor

	Total	Without complication	With complication	
Variable*	*n* = 95 (%)	*n* = 54 (%)	*n* = 41 (%)	*p*-value
Age, years (IQR)	57 (48 ~ 67)	57 (49 ~ 67)	57 (46 ~ 66)	0.974
Age ≥ 60 years	41 (43)	24 (44)	17 (42)	0.771
Male	52 (55)	26 (48)	26 (63)	0.139
mFI-5 score				0.366
< 2	60 (63)	32 (34)	28 (29)	
≥ 2	35 (37)	22 (23)	13 (14)	
Preoperative GCS (IQR)	15 (15 ~ 15)	15 (15 ~ 15)	15 (13 ~ 15)	-
Preoperative seizure	6 (6)	4 (7)	2 (5)	0.616
ASA score > 2	53 (56)	24 (44)	29 (71)	0.011
Preoperative laboratory data (IQR)				
WBC (K/µL)	9.0 (7.0 ~ 12.0)	8.5 (7.0 ~ 10.0)	9.0 (7.5 ~ 15.5)	0.059
Hct (%)	41.0 (37.0 ~ 44.0)	41.0 (37.0 ~ 44.0)	41.0 (36.5 ~ 44.0)	0.884
Hb (g/dL)	14.0 (12.0 ~ 15.0)	14.0 (12.0 ~ 15.0)	14.0 (12.0 ~ 15.0)	0.940
Platelet (K/µL)	218 (186 ~ 281)	231 (190 ~ 286)	209 (183 ~ 275)	0.249
Cr (mg/dL)	1.0 (1.0 ~ 1.0)	1.0 (1.0 ~ 1.0)	1.0 (1.0 ~ 1.0)	0.808
BUN (mg/dL)	15 (12 ~ 19)	14 (12 ~ 18)	15 (12 ~ 22)	0.190
eGFR (ml/min) (SD)	84.9 ± 23.3	86.7 ± 21.0	82.7 ± 26.1	0.409
Na (mmol/L)	141 (138 ~ 142)	141 (138 ~ 142)	140 (138 ~ 142)	0.533
K (mmol/L)	4.0 (4.0 ~ 4.0)	4.0 (4.0 ~ 4.0)	4.0 (4.0 ~ 4.0)	0.140
GPT (U/L)	19 (15 ~ 33)	19 (14 ~ 31)	21 (16 ~ 40)	0.614
Pathological type				0.108
Glioblastomas	70 (73.7)	41 (43.2)	29 (30.5)	
Anaplastic astrocytomas	10 (10.5)	3 (3.2)	7 (7.4)	
Anaplastic oligodendrogliomas	8 (8.4)	7 (7.4)	1 (1.1)	
Others	7 (7.4)	4 (4.2)	3 (3.2)	
WHO grade				0.972
3	23 (24)	13 (14)	10 (11)	
4	72 (76)	41 (43)	31 (33)	
Duration of anesthesia (h) (IQR)	11.0 (9.0 ~ 13.0)	10.0 (8.0 ~ 13.0)	12.0 (9.5 ~ 14.5)	0.221
Tumor volume (cm^3^) (SD)	6.7 ± 1.7	6.6 ± 1.6	6.7 ± 1.7	0.642

^*^Continuous data were tested for normality using the Kolmogorov–Smirnov test. Parametric continuous variables were presented as mean and standard deviation, and nonparametric data were presented as median and interquartile range. Abbreviations: *SD* Standard deviation, *IQR* Interquartile range, *mFI-5* Modified 5-item frailty index, *WHO* World Health Organization, *GCS* Glasgow coma scale, *ASA* American Society of Anesthesiologists, *WBC* White blood cells count, *Hct* Hematocrit, *Hb* Hemoglobin, *Cr* Creatinine, *BUN* Blood urea nitrogen, *eGFR* Estimated glomerular filtration rate, *Na* Sodium, *K* Potassium, *GPT* Alanine aminotransferase, *SBP* Systolic blood pressure, *DBP* Diastolic blood pressure

**Table 2:** Severity and types of complications of patients who underwent craniotomy for primary malignant brain tumor

Variable	Total *n* = 95 (%)
**Landriel classification grading, *n* (%)**	
No complications	54 (56.8)
Grade 1 (non-life-threatening)	23 (24.2)
Grade 2 (requires invasive treatment)	4 (4.2)
Grade 3 (life-threatening, ICU management)	12 (12.6)
Grade 4 (death)	2 (2.1)
**Complications, *n* (%)**	
**Surgical**	
Grade 1	
Transient new neurological deficit	4 (4.2)
Acute urinary retention	2 (2.1)
CSF infection requiring antibiotics	2 (2.1)
Sinus thrombosis requiring anticoagulation	1 (1.1)
Grade 2	
Postoperative infection with craniectomy	1 (1.1)
Dura defect with duraplasty	1 (1.1)
Grade 3	
ICH requiring reoperation	3 (3.1)
Acute hydrocephalus requiring EVD	2 (2.1)
Acute cerebral swelling requiring intubation	1 (1.1)
**Medical**	
Grade 1	
UTI with antibiotic treatment	6 (6.3)
Pneumonia with antibiotic treatment	6 (6.3)
Seizure with anticonvulsants	2 (2.1)
Grade 2	
Thrombectomy of right femoral vein thrombosis	1 (1.1)
Perianal ulceration with abscess formation	1 (1.1)
Grade 3	
Lung failure requiring intubation	5 (5.2)
ARDS	1 (1.1)
Grade 4	
Expired	2 (2.1)

Abbreviations: *ICU* Intensive care unit, *CSF* Cerebrospinal fluid, *ICH* Intracerebral hemorrhage, *EVD* External ventricular drain, *UTI* Urinary tract infection, *ARDS* Adult respiratory distress syndrome

**Table 3:** Perioperative mean change in serum laboratory parameters of patients who underwent craniotomy for primary malignant brain tumor

	Total	Without complication	With complication	
Variable (mean ± SD)	*n* = 95 (%)	*n* = 54 (%)	*n* = 41 (%)	*p*-value
WBC (K/µL)	4.7 ± 5.6	5.8 ± 5.1	3.3 ± 6.1	0.031
Hct (%)	− 4.8 ± 4.1	− 5.4 ± 3.5	− 4.1 ± 4.6	0.138
Hb (g/dL)	− 1.6 ± 1.4	− 1.7 ± 1.2	− 1.5 ± 1.7	0.390
Platelet (K/µL)	− 33.7 ± 42.9	− 34.2 ± 33.4	− 33.1 ± 53.3	0.904
Cr (mg/dL)	− 0.1 ± 0.2	− 0.1 ± 0.3	0.0 ± 0.2	0.620
BUN (mg/dL)	− 0.3 ± 5.8	− 1.0 ± 5.5	0.6 ± 6.2	0.184
eGFR (ml/min)	13.3 ± 27.9	16.8 ± 30.9	8.8 ± 23.1	0.167
Na (mmol/L)	1.2 ± 4.7	0.7 ± 3.9	1.8 ± 5.6	0.254
K (mmol/L)	− 0.2 ± 0.7	− 0.2 ± 0.6	− 0.1 ± 0.8	0.885
GPT (U/L)	0.1 ± 30.1	2.4 ± 19.7	− 3.0 ± 40.0	0.382
KeGFR (mL/min)	− 0.4 ± 6.7	0.9 ± 6.3	− 2.0 ± 7.1	0.041

Abbreviations: *SD* Standard deviation, *WBC* White blood cells count, *Hct* Hematocrit, *Hb* Hemoglobin, *Cr* Creatinine, *BUN* Blood urea nitrogen, *eGFR* Estimated glomerular filtration rate, *Na* Sodium, *K* Potassium, *GPT* Alanine aminotransferase, *KeGFR* Kinetic estimated glomerular filtration rate

### Significant association between higher ASA physical status score, longer anesthesia duration, and decreased KeGFR and hematocrit values with PC1M

In multivariable logistic regression analysis, of six potential variables, one preoperative physical factor, one intraoperative surgical metric, and two serum laboratory parameters differed significantly between the groups: an ASA physical status score of > 2 (adjusted odds ratio [aOR], 3.17; 95% confidence interval [CI], 1.19–8.45; *p* = 0.021), anesthesia duration (*aOR*, 1.16; 95% *CI*, 0.69–0.88; *p* < 0.001), perioperative change in Hct by >  − 4.8% (*aOR*, 3.45; 95% *CI*, 1.22–9.73; *p* = 0.0019), and perioperative change in KeGFR of < 0 mL/min (*aOR*, 3.99; 95% *CI*, 1.52–10.53; *p* = 0.005). The combination of these four parameters created an accurate predictive model of PC1M, with an area under the curve of 0.780 (95% *CI*, 0.69–0.88; Table 4).

**Table 4:** Factors independently associated with postoperative complication using logistic regression

	Univariate		Multivariate	
Variable	OR (95% *CI*)	*p*-value	aOR (95% *CI*)	*p*-value
Age ≥ 60 years	0.89 (0.39–2.01)	0.771		
Male	1.89 (0.81–4.28)	0.140		
mFI-5 score > 1	0.60 (0.25–1.43)	0.250		
Tumor pathological type	1.14 (0.91–1.41)	0.250		
WHO grade 4	0.98 (0.38–2.54)	0.972		
Tumor volume (cm^3^)	1.14 (0.91–1.41)	0.250		
Preoperative GCS	0.77 (0.62–0.96)	0.018		
Preoperative seizure	0.64 (0.11–3.68)	0.618		
ASA score > 2	3.02 (1.28–7.14)	0.012	3.17 (1.19–8.45)	0.021
ΔWBC > 4.7 (K/µL)	0.64 (0.28–1.46)	0.288		
ΔHct > − 4.8 (%)	2.41 (1.04–5.58)	0.040	3.45 (1.22–9.73)	0.019
ΔHb > − 1.6 (g/dL)	1.68 (0.74–3.82)	0.212		
ΔPlatelet > − 33 (K/µL)	1.13 (0.50–2.57)	0.771		
ΔCr > − 0.1 (mg/dL)	1.56 (0.27–8.96)	0.618		
ΔBUN > − 0.3 (mg/dL)	2.05 (0.88–4.79)	0.097		
ΔNa > 1.2 (mmol/L)	1.26 (0.55–2.85)	0.585		
ΔK > 0 (mmol/L)	1.68 (0.48–5.94)	0.421		
ΔGPT > 0 (U/L)	1.05 (0.47–2.37)	0.906		
ΔKeGFR < 0 (mL/min)	3.03 (1.30–7.06)	0.010	3.99 (1.52–10.53)	0.005
Duration of anesthesia (h)	1.09 (1.00–1.19)	0.062	1.16 (1.04–1.29)	0.009

Abbreviations *SD* Standard deviation, *mFI-5* Modified 5-item frailty index, *WHO* World Health Organization, *GCS* Glasgow coma scale, *ASA* American Society of Anesthesiologists, *WBC* White blood cells count, *Hct* Hematocrit, *Hb* Hemoglobin, *Cr* Creatinine, *BUN* Blood urea nitrogen, *Na* Sodium, *K* Potassium, *GPT* Alanine aminotransferase, *KeGFR* Kinetic estimated glomerular filtration rate

### Older age as a significant subgroup in the KeGFR model

In the group of patients older than 65 years, the analysis revealed a significantly heightened risk for PC1M in the KeGFR model (*OR*, 11.55; 95% *CI*, 1.30–102.79; *p* = 0.028; postoperative KeGFR—preoperative estimated GFR obtained using the MDRD equation; Table 5). Furthermore, although there was an observed elevated risk in the male group, it merely presented a trend toward statistical significance (*OR*, 3.80; 95% *CI*, 0.95–15.12; *p* = 0.059).

**Table 5:** Stratification analysis for the risk of postoperative complication in the KeGFR model

Variables	aOR (95% *CI*)	*p*-value
Sex		
Male	3.80 (0.95–15.12)	0.059
Female	3.62 (0.81–16.15)	0.092
Age		
≤ 50 years	7.48 (0.67–84.17)	0.103
50–65 years	1.90 (0.42–8.69)	0.406
≥ 65 years	11.55 (1.30–102.79)	0.028

Abbreviations: *KeGFR* Kinetic estimated glomerular filtration rate, *MDRD* Modification of diet in renal disease, *aOR* Adjusted odds ratio, *CI* Confidence interval

## Discussion

We identified four significant parameters of PC1M in patients who had undergone craniotomy for PMBT removal: worse ASA physical status score, longer anesthesia duration, and decreased perioperative Hct and KeGFR values. The well-validated KeGFR equation with volume change and fluid balance was first applied to evaluate renal clearance change following neurosurgery. Furthermore, stratification analysis revealed that the age of ≥ 65 years and male sex were the more robust subgroups associated with PC1M.

### Various classification systems for PC1M

Several classification systems were developed to provide information regarding the severity of postoperative complications (Bonsanto M. M. et al., 2001; Houkin K. et al., 2009). Landriel classification was modified from the classification of complications by Clavien and Dindo to match the need of neurosurgical patients (Manekk et al. 2022). Currently, the Landriel classification is the most accepted and practiced in neurosurgery based on the interventions required to manage the complications (Gómez Vecchio et al. 2021). This is the simplest form of classification that can be used to categorize surgical complications and is easily understood by medical and paramedical staff. However, Ferroli et al. indicated that these classifications, which are treatment based, do not provide the full complexity of patient and disease characteristics (Ferroli et al. 2014). From this perspective, Chandra Venkata Vemula et al. created a modified Landriel classification incorporating the HRQoL score (Chandra Venkata Vemula et al., 2021). The scale aimed to appropriately measure the outcome of surgery related to the quality of life of patients. In our opinion, the KeGFR changes represented the acute physiological changes in renal function but did not predict the long-term variations in the quality of life. Hence, we used the original Landriel classification in this study.

### Associations of specific physical and surgical metric with PC1M

We confirmed that a higher ASA physical status score was a significant risk factor for PC1M. This score reflects the general physical status of patients, thereby represnting the ability of the patients to respond to a disease or surgery burden; higher scores are associated with poor outcomes (Saklad 1941). ASA physical status scores of > 2 in patients who have underfone neurosurgery are associated with PC1M, poorer short-term outcomes, and longer hospitalization (Dasenbrock et al. 2015; Senders et al. 2018). Thus, this score can be used as a practical preoperative tool to identify patients with PMBTs who are at risk for PC1M.

A longer anesthesia duration was also associated with a higher incidence of PC1M in patients with PMBTs. Under the definition, the time duration partially represents the period during which the patient is under the influence of anesthetic drugs. Prolonged anesthesia duration in several types of surgery could lead to PC1M; however, to the best of our knowledge, no objective evidence in neurosurgery has been reported (Brady et al. 2018; Phan et al. 2017). Anesthesia duration is the sum of the time involved in anesthesia induction, surgery, and emergence; thus, it reflects antiseptic preparation and surgical complexity. Compared with other operations, the complexity of intracranial surgery contributes to its long duration, and the more significant anesthetic side effects of the related sedative drugs may also be associated with PC1M development (Schubert et al. 1996). Our results revealed that prolonged anesthesia duration independently associates with the overall PC1M. Therefore, shortening the duration of induction and antiseptic preparation may be as crucial as reducing surgical time.

### Association of perioperative change in hematocrit levels with PC1M

Our findings suggest that a decline in Hct levels of > 4.8% is related to PC1M development in patients with PMBTs. Decreased Hct levels represent the severity of the effects of insufficient oxygen supply to the brain and anemic hypoxia (Kiyohara et al. 1985). Lower postoperative hematocrit levels increase the risk of complications and poor neurological outcomes in patients with critical illnesses, those undergoing craniectomy for traumatic brain injury (TBI), and those undergoing major noncardiac surgery (Rajagopalan et al. 2019; Wu et al. 2007). Zhou et al. reported that both lower postoperative and sizable decline in Hct levels can predict poor short-term prognosis of a patient with TBI (Zhou et al. 2019).

### Brain–kidney interaction and perioperative change in KeGFR

In brain research, several reports have demonstrated the brain–kidney interaction in stroke and TBI. A major phenomenon in adult patients with neurological injuries is augmented renal clearance (ARC), defined as 24-h CrCl of > 130 mL/min/1.73 m^2^. The dominant theory is that ARC is a consequence of the reaction of the body to critical illness and fluid resuscitation therapy, both of which increase cardiac output and, therefore, circulation through the kidneys (Dias et al. 2015). ARC is the physiological response of the kidneys to various stimuli and is considered to result from excessive use of the renal reserve. Reducing renal clearance is strongly associated with PC1M in patients who underwent craniotomy for PMBT removal. The relationship between ARC and glomerular function has been increasingly reported in studies regarding neurocritical care for intracranial hemorrhage, TBI, and postneurosurgical status (Udy et al. 2017). ARC might represent the recruitment of the renal system of the physiological reserve of glomerular filtration when systemic biological stress occurs following neurosurgery (Cook & Hatton-Kolpek 2019). The lack of a backup kidney filtration, which would lead to stagnant or even decreased renal clearance, would significantly enhance the risk of PC1M.

ARC has been associated with elevated urinary CrCl levels. The GFR is generally accepted as the best overall index of kidney function for determining ARC presence (Bilbao-Meseguer et al. 2018). Although urine creatinine tests are probably more accurate predictors of kidney injury, they are not yet routinely conducted for postoperative critically ill patients. Serum creatinine concentration is a relatively insensitive marker of GFR but is a commonly used surrogate measure of renal function (Udy et al. 2013). Therefore, several mathematical equations based on serum creatinine concentration are used to estimate GFR. In the equations currently used to estimate GFR, renal function can be estimated when the serum creatinine level is stable. However, these equations do not yield accurate results if the serum creatinine level and fluid balance change rapidly. Acute changes in renal clearance during the postoperative period might result from the volume distribution of body fluid, which is affected by intravenous fluid administration, and from the variation in cardiac output due to perioperative anesthesia effects and physiological stress (Baptista et al. 2019). The KeGFR equation provides clinically relevant real-time values of GFR without requiring periodic urine collections (Chen 2013). We have used an equation for calculating the “kinetic” estimated GFR to determine CrCl levels or GFR in the nonsteady state, wherein two creatinine measurements and fluid balance are used. Herein, 24-h postoperative renal clearance was increased in both nonPC1M and PC1M groups in the MDRD equation group. Conversely, the values calculated using the KeGFR equation indicated ARC presence in the nonPC1M group and a 24-h postoperative decrease in renal clearance in the PC1M group. Our results revealed that the KeGFR equation enabled a more sensitive detection of a slight decline in renal function following neurosurgery than the MDRD equation. Several similar applications of KeGFR have already been used extensively in clinical research and have yielded accurate results (Pianta et al. 2015; Seelhammer et al. 2016). Thus, the KeGFR equation helps identify patients at risk for subsequent renal clearance impairment (Chen & Chiaramonte 2019).

### Older age was a robust subgroup in associations with PC1M

Furthermore, our stratification analysis revealed a heightened risk of PC1M in older patients (aged ≥ 65 years) according to the KeGFR equation. Simultaneously, a trend toward an increased risk of PC1M was discernible among male patients, yet this did not attain statistical significance, which could potentially be attributed to the limited sample size of our study. This result also coincided with previous findings that ARC may be a common phenomenon, particularly among young and previously healthy patients undergoing neurosurgery (Chen et al. 2020). ARC is associated with trauma, young age (< 50 years), male sex, and lower score on the Sequential Organ Failure Assessment (Wu et al. 2019). Age and sex may affect the brain–kidney interaction, which was also described in patients with ischemic and hemorrhagic stroke (Zhao et al. 2020). As noted previously, the relative deficiency in alternative renal filtration in older patients increases the possibility of PC1M. Therefore, this factor, which is more common than the expected risk, should be monitored in neurosurgery departments to ensure early treatment.

### Limitations

This study had certain limitations. First, the KeGFR has been effective in various clinical contexts (Chen & Chiaramonte 2019; Chen et al. 2020); its validation in neurosurgical patients remains to be done. The creatinine data, obtained from 7 days pre to 24-h post-operation, may exceed the recommended 72-h time difference, potentially affecting estimation accuracy. Despite these constraints, our patients’ good preoperative health (ASA scores 2–4), suggesting a reasonable assumption of preoperative creatinine value stability. Secondly, despite the small sample size, power analyses, based on KeGFR and other key factors, yielded substantial values (*KeGFR*: 0.881; anesthesia duration: 0.850; *Hct*: 0.818; ASA score: 0.735). These figures underscore the robustness of our findings and suggest that the KeGFR equation can identify patients at risk for PC1M early, even in nonoptimized cohorts. Third, we evaluated only PMBT by discerning the tumors’ histological type. However, no molecular markers and lesion sites were analyzed, potentially limiting the generalizability of our findings. Lastly, due to the constraints inherent in a retrospective study design, including a lack of detailed hospital records regarding anethesia time segments, further prospective studies focusing on renal clearance changes are necessary for a more comprehensive understanding of the postoperative changes in patients undergoing neurosurgery.

## Conclusions

Our research identified four key parameters—higher ASA physical status score, longer anesthesia duration, and perioperative changes in KeGFR and Hct values—that appear linked to the occurrence of PC1M. Furthermore, we conducted comparative and descriptive analyses of perioperative changes in renal function using different equations for calculating GFR. KeGFR emerged as an effective tool for discerning patients who, minimal changes in perioperative creatinine or eGFR, may have hidden risks for complications. Consequently, the incorporation of keGFR evaluation is recommended in the postoperative management of patients undergoing primary malignant brain tumor resection, particularly among the elderly population.

## Data Availability

The datasets used and/or analyzed during the current study are available from the corresponding author on reasonable request.

## Abbreviations

AKI: Acute kidney injury
ARC: Augmented renal clearance
ASA: American Society of Anesthesiologists
GCS: Glasgow Coma Scale
GFR: Glomerular filtration rate
KeGFR: Kinetic estimated glomerular filtration rate
KVGH: Kaohsiung Veterans General Hospital
MDRD: Modification of Diet in Renal Disease
PC1M: Postoperative complications within 30 days
PMBT: Primary malignant brain tumor
SHR: Spontaneously hypertensive rats
TBI: Traumatic brain injury
WBC: White blood cell
WHO: World Health Organization
